# Adolescent-Acquired Flatfeet: The Tip of the Iceberg

**DOI:** 10.7759/cureus.30983

**Published:** 2022-11-01

**Authors:** Tahani Al Ali, Sattar Alshryda

**Affiliations:** 1 Pediatric Orthopaedics and Trauma, Al Jalila Children's Speciality Hospital, Dubai, ARE; 2 Trauma and Orthopaedics, Mohammed Bin Rashid University of Medicine and Health Sciences, Dubai, ARE

**Keywords:** covid-19, muscle length, limb deformity, prevention review, flat foot

## Abstract

There has been a substantive change in our lifestyle over the last two decades. The widespread availability of entertaining digital devices created an unhealthy culture of a sedentary lifestyle, with our children hooked to their digital devices for countless hours. The mental and social consequences have been well explored in several studies. Leading a sedentary lifestyle has been shown to be associated with obesity, diabetes, cardiovascular diseases, and even early death. The adolescent-acquired flatfeet is another addition to the ever-growing list.

The lack of physical activities among children nowadays has led to a pandemic of long muscles tightness in children, particularly during the growth spurt. The mismatch between the long bones and adjacent muscles growth caused relative muscles shortening, particularly the muscles that cross more than one growth center, such as the hamstring muscles and gastrocnemius muscles. As a result, it has become common to see children who cannot touch the floor on forward bending because of hamstring muscles tightness or inability to walk on their heels because of gastrocnemius muscles tightness. While muscles tightness is relatively benign, its consequences, such as adolescent-acquired flatfeet, are not. In this review, we have explored the condition, its prevention, and treatment to raise awareness among the public and professionals.

## Introduction and background

There has been a substantive change in children's lifestyles over the last 20 years which was triggered by the introduction of hundreds of digital gadgets and online games. Children spend countless hours on their tablets, computers, PlayStation®, Xbox, and many other digital entertaining devices. Some get addicted to using these devices [1,2]. This became worse during the COVID-19 pandemic and subsequent lockdowns [3]. The introduction of home learning during the lockdown period slackens the parental restriction on the usage of digital devices among children because it has become difficult for parents to differentiate between educational and entertainment usage. As a result, children's sports and social activities have dropped significantly. Until recently, parents often struggled to bring their children from playgrounds to home; now, we witness the reverse when parents struggle to get their children to play outside the house. Unfortunately, this came with a hefty price when many children develop muscles tightness and subsequently acquired flatfeet - a deformity that was seen only in children with cerebral palsy or children with poor mobility [4]. The surge in the number of patients that we see in our clinic and operate on is alarming, and this is just the tip of the iceberg. Public awareness is essential to safeguard our children.

## Review

**Figure 1:**
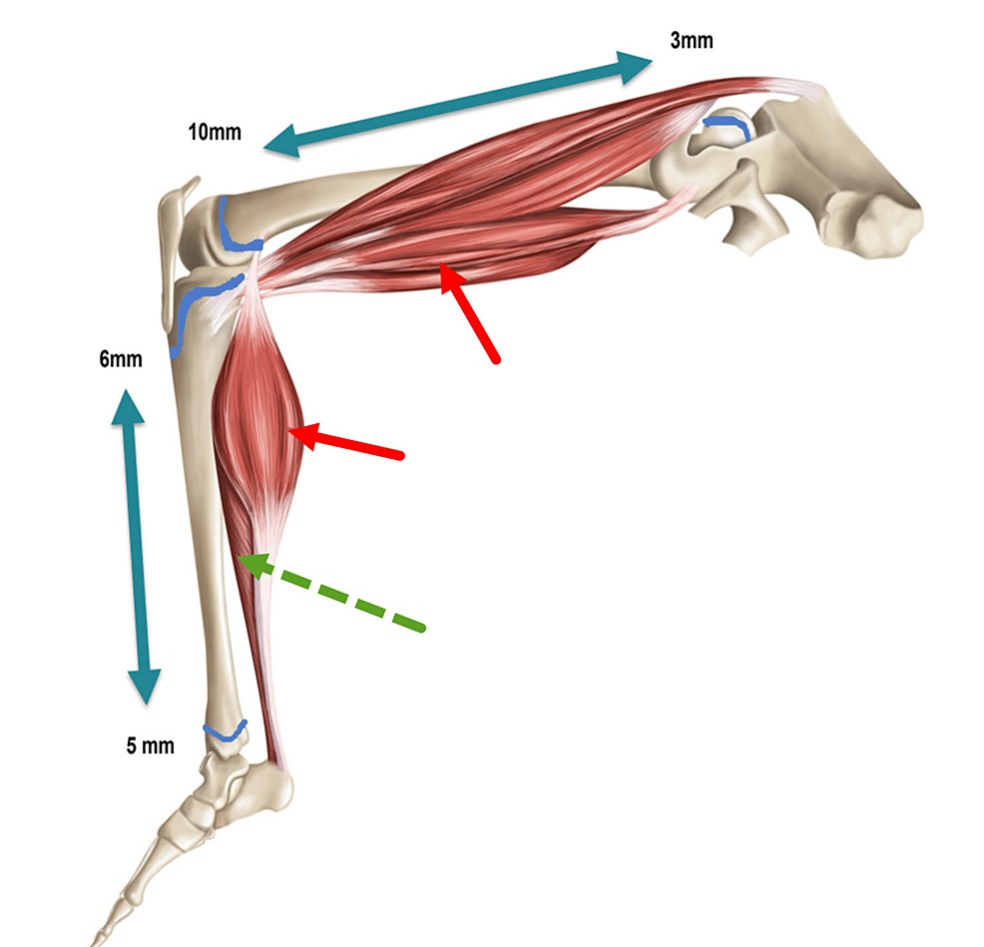
A diagram shows the lower limb bones and key muscles The bones grow longer through the growth centers. The muscles get stretched to match the adjacent bones' length when children play and run. This muscle stretching does not happen in sedentary children who end up with relatively shorter muscles. The muscles that cross multiple growth centers, such as the hamstring and gastrocnemius muscles (red arrows), are more affected than the muscles that cross one growth center, such as the soleus (dashed green arrow). Redrawn from [6] with permission.

Bones in children grow longer through growth centers (also called physes). There are two growth centers in each long bone. One is at the top, and another is at the bottom. For example, the proximal growth center of the tibia grows 6 mm per year, whereas the distal growth center grows 5 mm per year. This means that in an average child, the tibia gets longer by 11 mm per year. Six mm is contributed by the proximal growth center, and 5 mm is contributed by the distal growth center. Similarly, the femur (or the thigh bone) grows 14 mm per year, 10 mm from the distal growth center and 3 mm from the proximal growth center (Figure 1) [5].

**Figure 2:**
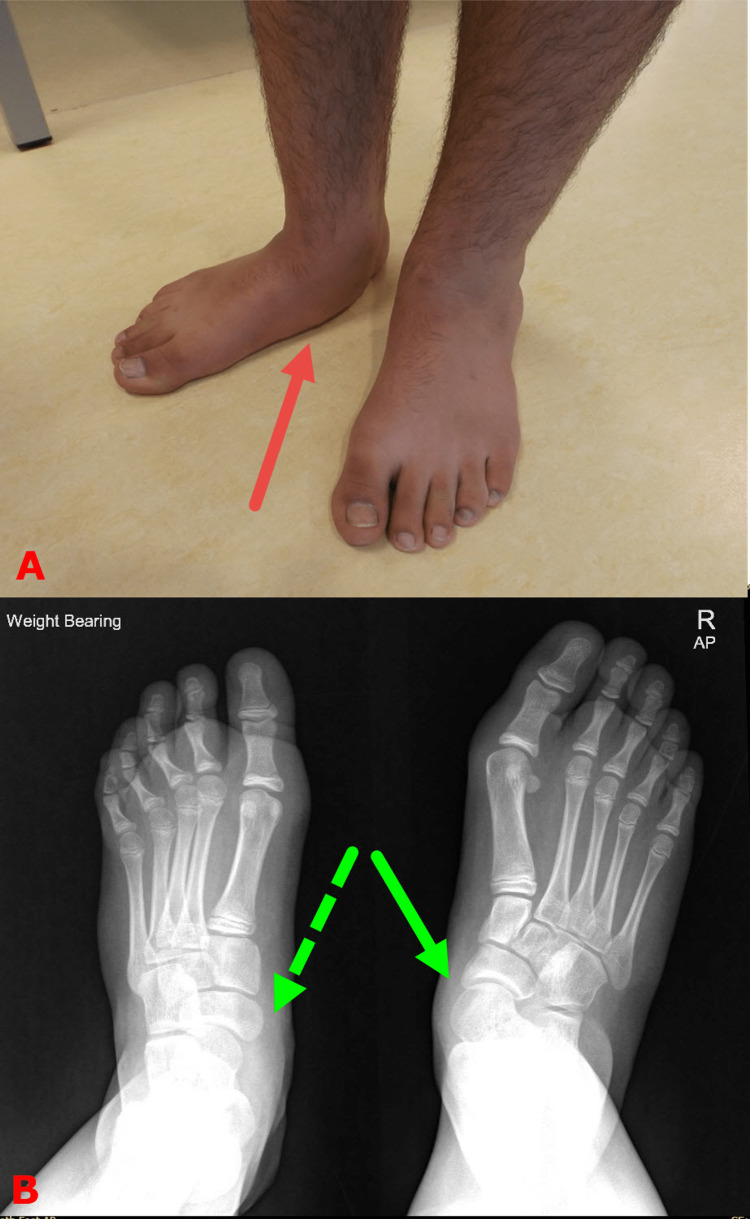
Clinical photograph and plain X-ray of the feet of a child with acquired adolescent flatfoot Top image (A) is a clinical photograph of a child who developed an adolescent flatfoot on the right side. Notice the loss of the medial longitudinal arch (red arrow). Bottom image (B) is a plain radiograph of weight-bearing feet. Notice the subluxated right talonavicular joint (solid green arrow) in comparison to the left side (dashed green arrow). Images are provided by the authors.

For optimal function, muscles have to grow to match the length of adjacent bones. Muscles, which are made of sliding fibers, get longer by stretching, which happens during children's daily activities such as walking, running, and playing. Children who adopt a sedentary lifestyle would eventually have shorter muscles. This is clinically manifested as a bouncy gait or toe walker because of the tight gastrocnemius muscles. This is considered stage I of the condition. As children get older and heavier, the condition gets worse. The small joints of the foot, particularly the talonavicular joint, succumb under the heavy weight and start dislocating. This clinically manifests itself by losing the already developed medial longitudinal arch of the foot (Figure 2). This denotes stage II of the condition. There is no pain in stage I and early stage II. However, as the talonavicular joint dislocates more, the talus head uncovering increases, and the body weight distributes to a relatively smaller area initiating pain on walking and tip-toeing. Pain heralds the third stage of the condition.

**Figure 3:**
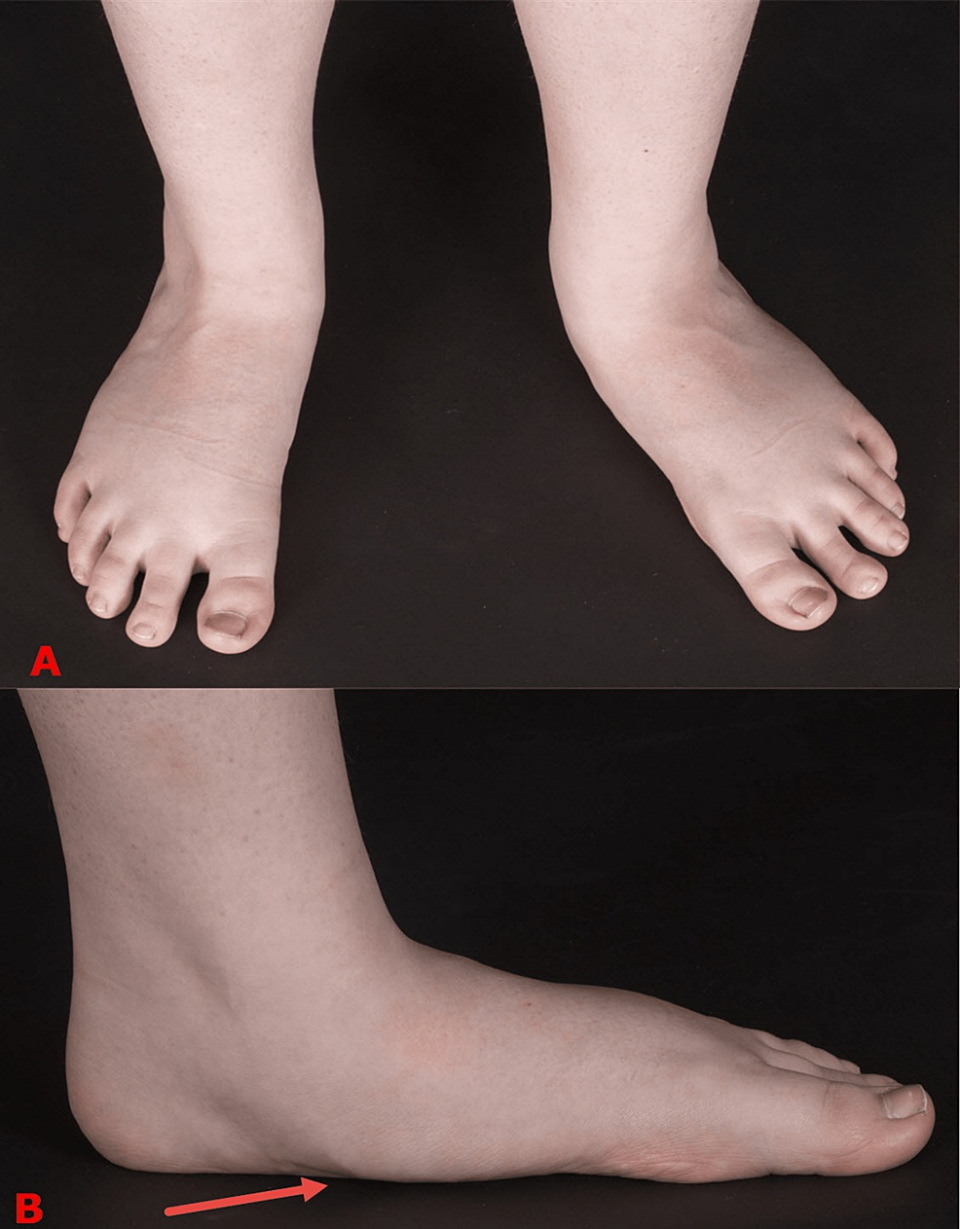
Clinical photograph of the feet of a child with adolescent-acquired flatfoot The loss of the medial arch and the outward-facing foot are common presentations of adolescent-acquired flatfoot. The top image (A) shows the left foot pointing outward in comparison to the right foot. The bottom image (B) shows that the medial longitudinal arch of the foot has collapsed. This usually indicates that the talonavicular joint has subluxated (red arrow). Images are provided by the authors.

**Figure 4:**
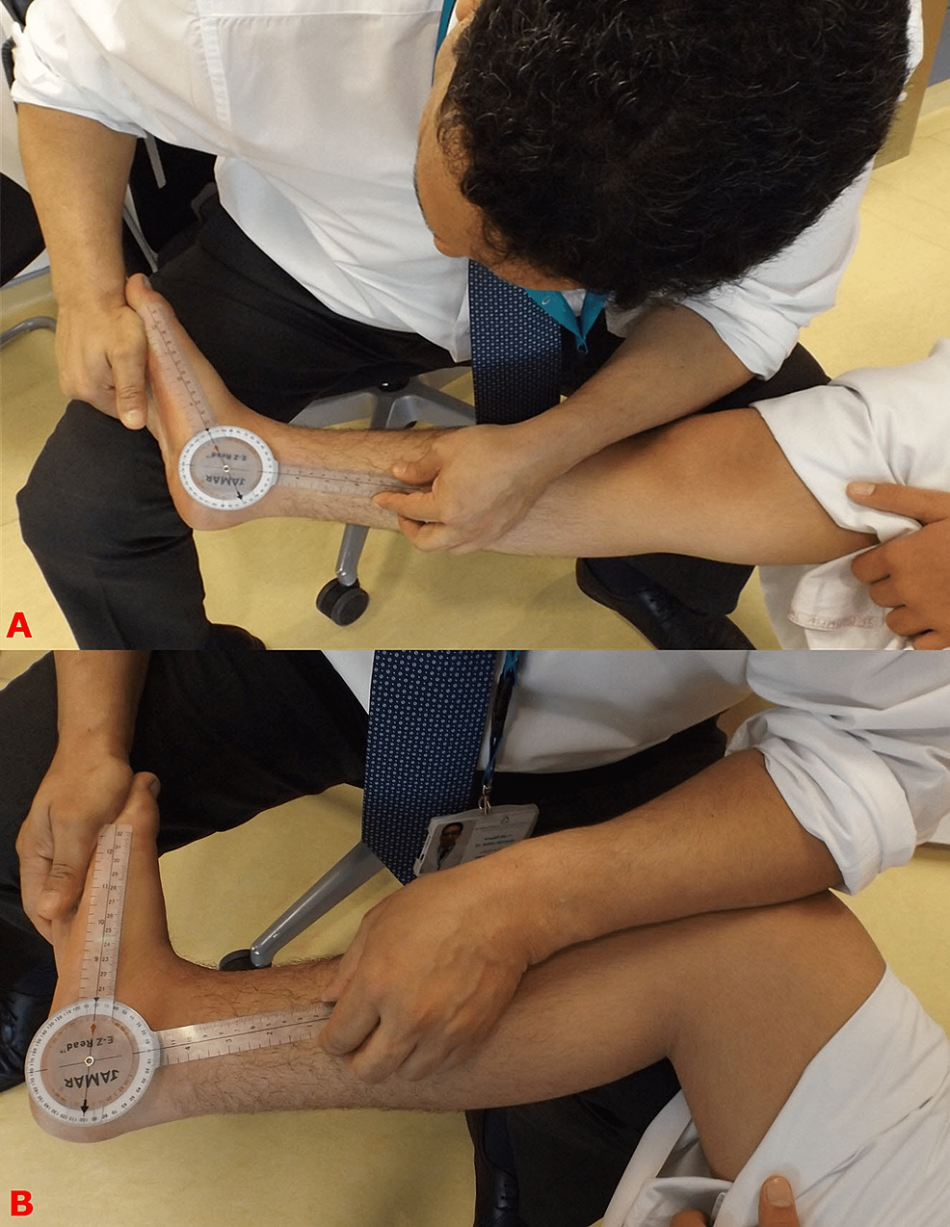
Clinical photograph demonstrating gastrocnemius muscle tightness Silverskiöld test to differentiate gastrocnemius muscles tightness and soleus muscle tightness. Top image (A) shows that the ankle joint has an equinus contracture. This is because either the gastrocnemius, soleus, or both muscles are tight. Flexing the knee relaxes the gastrocnemius muscles and allows ankle dorsiflexion to a normal level if the soleus muscles are not tight, as is the case in this child (bottom image B). Images are provided by the authors.

Children with adolescent-acquired flatfeet are usually present between the age of 10-14, typically after a growth spurt. It starts on one foot, then the other side follows. The common presentation is the loss of the medial arch or the foot is pointing outward, which is relatively late for non-operative treatments to be effective (Figure 3). The active screening program in our center has been successful in identifying many children with stage I. In this stage, the foot is completely normal. There is tightness in the gastrocnemius muscles, but the soleus is often of normal length (Figure 4). Confirming muscle tightness is essential to differentiate adolescent-acquired flatfeet from developmental flatfeet, which is common in this age group [6,7].

**Figure 5:**
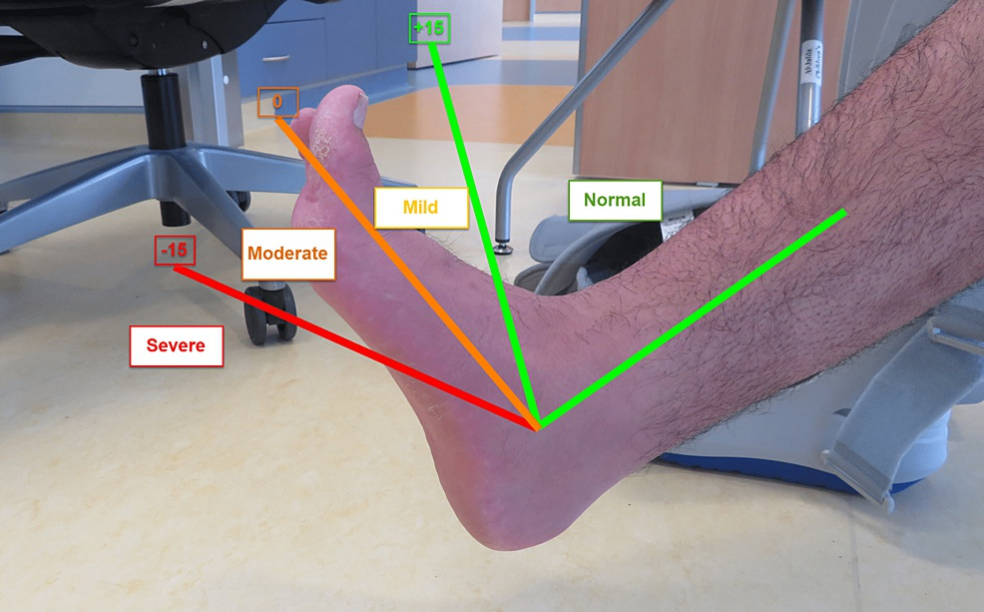
A diagram of equinus severity grading The severity of the muscle tightness is divided into three grades: mild tightness when ankle dorsiflexion is between 0° and +15°, moderate tightness when ankle dorsiflexion is between 0° and -15° and severe tightness when ankle dorsiflexion is <-15°. The image is provided by the authors.

We grade the severity of the muscle tightness into three grades. Mild tightness when ankle dorsiflexion is between 0° and +15°; moderate tightness when ankle dorsiflexion is between 0° and -15° and severe tightness when the ankle dorsiflexion is <-15° (Figure 5).

**Figure 6:**
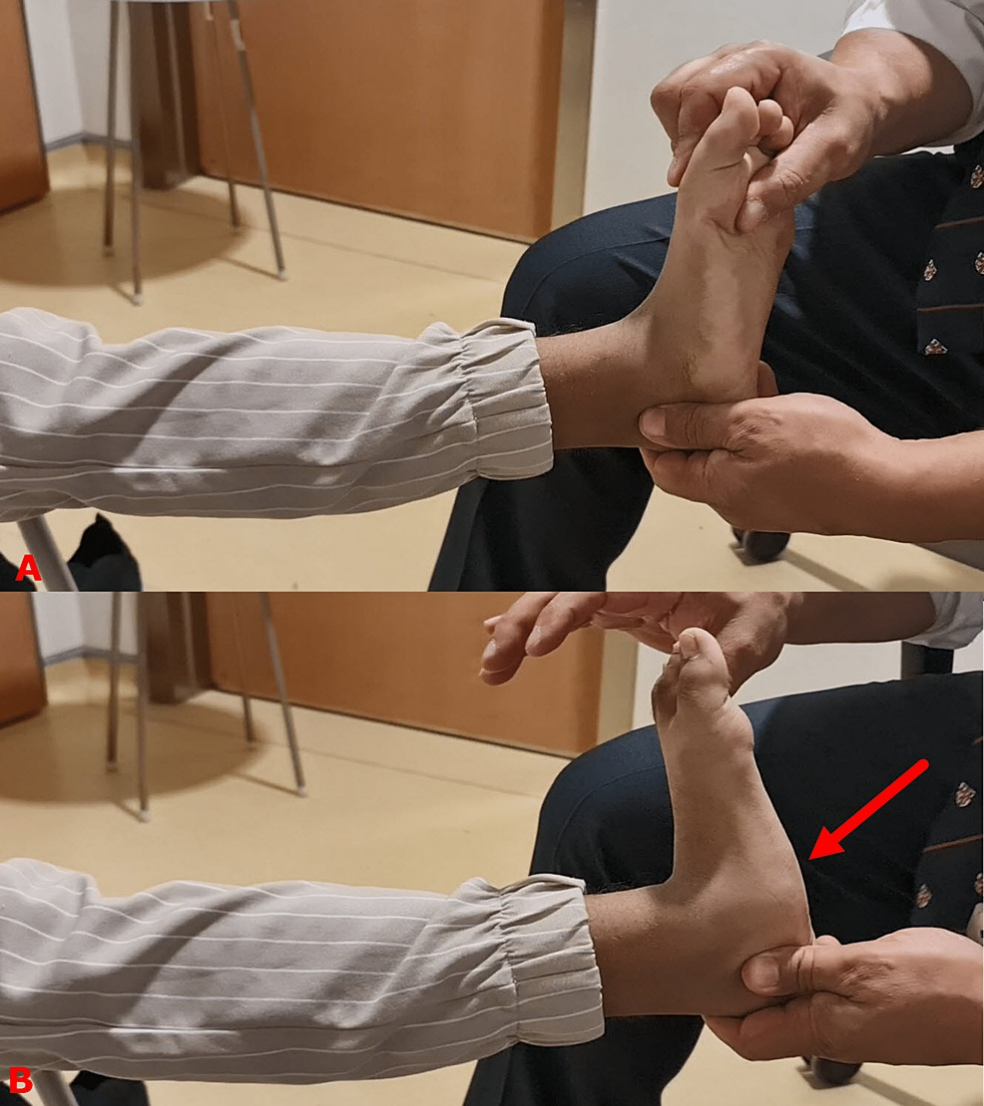
Clinical photograph demonstrating a common pitfall in assessing the severity of equinus contracture A common pitfall is the underestimation of the gastrocnemius muscle tightness when there is a subluxation of the talonavicular joint. The top image (A) shows the correct way to test for gastrocnemius muscle tightness by supinating the foot and locking the midfoot movement. The test shows moderate to severe tightness. The bottom image (B) shows the same foot being tested incorrectly. Although the position of the foot gives the impression that there is no muscle tightness, most of the movement happens in the midfoot joints (red arrow) and not in the ankle joint. Images are provided by the authors.

A common pitfall is the underestimation of the gastrocnemius muscle tightness when there is a subluxation of the talonavicular joint. The pathologically excessive movement of the joint masks muscle tightness by allowing the foot to be pushed more upward (Figure 6).

Children in stage I walk with a very characteristic bouncy gait. They have a normal initial heel contact at the beginning of the stance phase of the gait cycle (normal first rocker). Then the body moves normally forward on the planted foot until the gastrocnemius muscle tightness stops this forward movement of the body. At this stage, children have to raise the planted heel and go on their toes to maintain the forward body movement (shortened second rocker). This leads to the characteristic gait as if they bounce up and down on their toes in the middle of their walk.

When the tightness is more severe, children do not have heel strikes at all, and they present as toe walkers.

**Figure 7:**
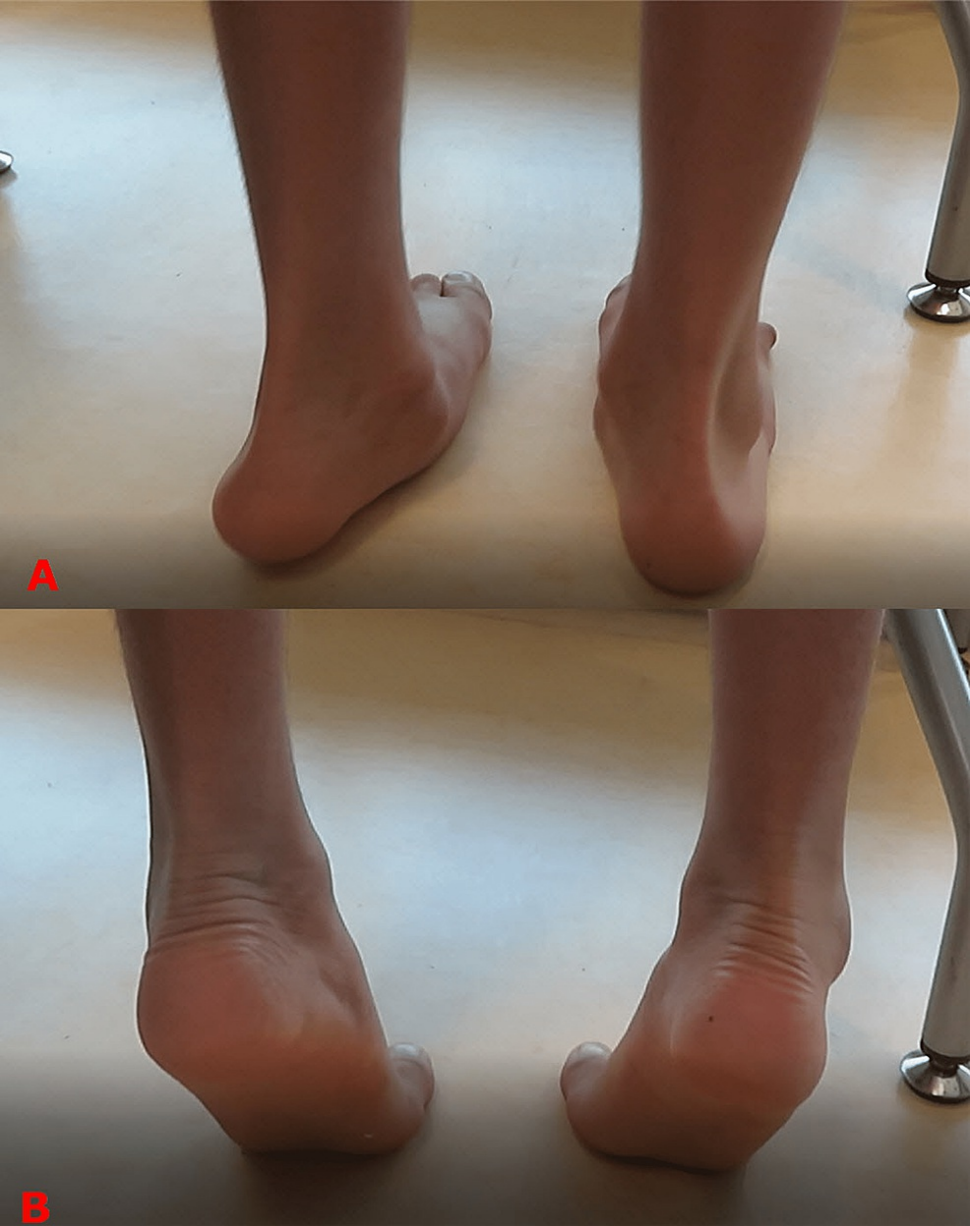
Clinical photographs showing the flexibility of the adolescent-acquired flatfeet Adolescent flatfeet remain flexible until late in the disorder's progress. During standing, both heels are in valgus, and the arches are flattened (top image A). However, on tip-toeing, the heels move into varus, and the arches get reconstituted (bottom image B). Images are provided by the authors.

The feet remain flexible until the advanced stages, a very important differentiating sign from the tarsal coalition and the adult acquired flatfeet [8]. By flexibility, we mean that heels moved into varus, and the medial arches are reconstituted on tip-toeing (Figure 7) [9,10,11].

**Figure 8:**
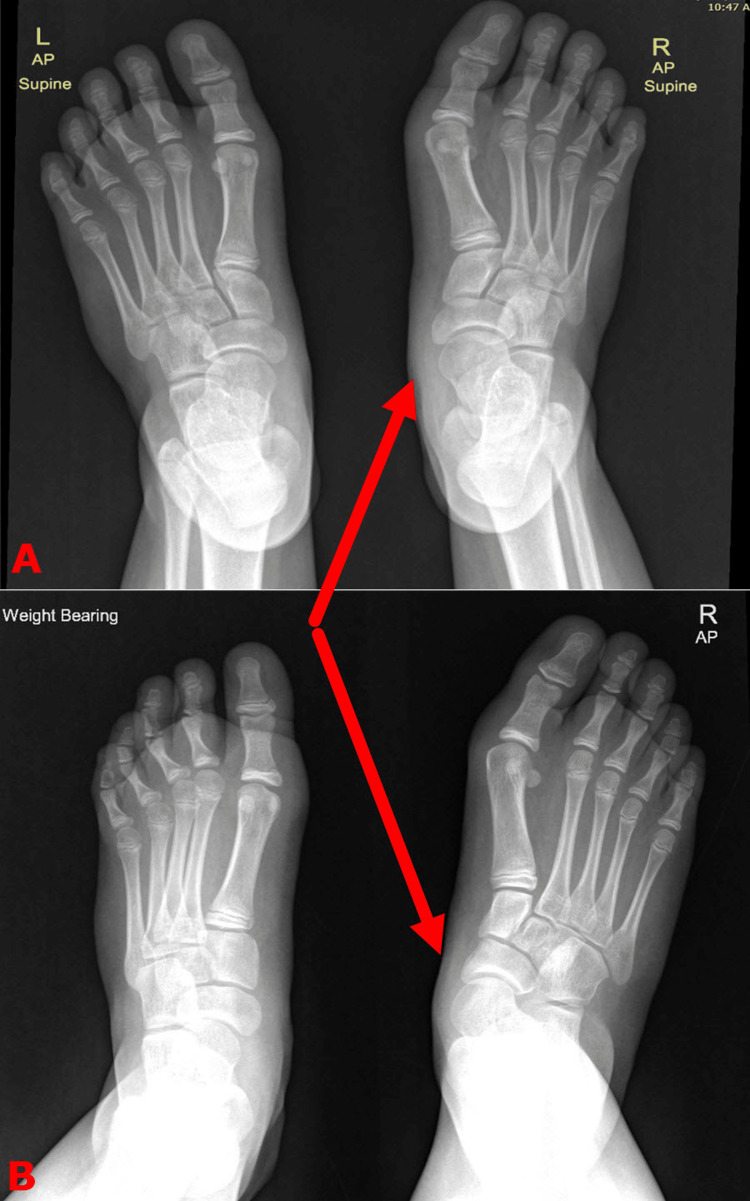
Plain radiographs of the same child reflecting the importance of weight-bearing views The supine (or non-weight-bearing) views of the feet X-rays (top image A) can mask the talonavicular joint subluxation (red arrows), which is more pronounced in the weight-bearing views (bottom image B). Images are provided by the authors.

A plain radiograph of the feet in a weight-bearing position is important to confirm the foot involvement and also to quantify the severity. Non-weight-bearing feet X-rays can be deceptively normal (Figure 8). 

A radiograph is normal in stage I of the disease. This means that the navicular bone is covering the talus head fully, and the angle between the first metatarsal bone and the main axis of the talus is within normal (10±7°) [12,13]. We consider the deformity mild when the angle is ≤30°, moderate when the angle is ≤45°, and severe when the angle is >45°.

**Figure 9:**
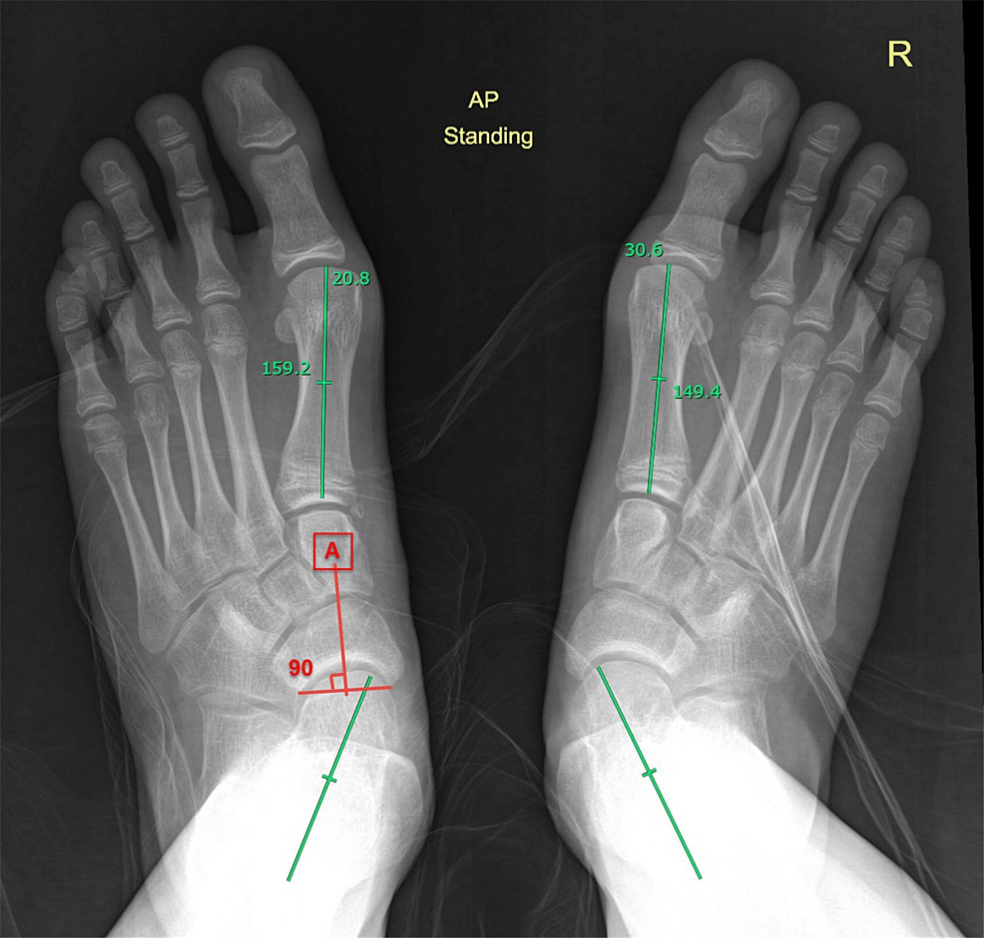
Plain radiograph of a child with adolescent-acquired flatfoot Weight-bearing feet radiograph shows bilateral mild talonavicular subluxation, mild on the left (the talo-first metatarsal angle is 20.8°) and moderate on the right (the angle is 30.6°; green lines). The red line (A) represents the navicular concave joint surface bisector line which can be used instead of the first metatarsal line when there is a coexisting metatarsus adductus. The image is provided by the authors.

The angle underestimates the magnitude of the deformity when a metatarsus adductus deformity coexists. The navicular concave joint surface bisector line is used instead of the first metatarsal line (Figure 9). CT or MRI scans are not normally indicated unless tarsal coalition or other foot pathologies are suspected.

Prevention is better than treatment. Public awareness of the condition and the importance of adopting a healthy lifestyle is key. This can be achieved at various levels. The involvement of governmental bodies can play an enormous role in preventing this condition and its financial impact on public health systems.

In the early stages of the condition, non-operative treatments can be very successful. Our first line of treatment is physiotherapy, particularly when the tightness is mild or moderate. In compliant children, mild tightness takes about three months of regular daily stretching exercises to return to normal. Moderate and severe tightness take about six and 12 months, respectively. While the tight muscles are getting stretched, we recommend medial arch support orthoses to protect the foot arches [14]. These are only temporary until the muscles get back to normal length. Most children in this age group are non-compliant and this is the main reason why physiotherapy treatment is not always successful.

**Figure 10:**
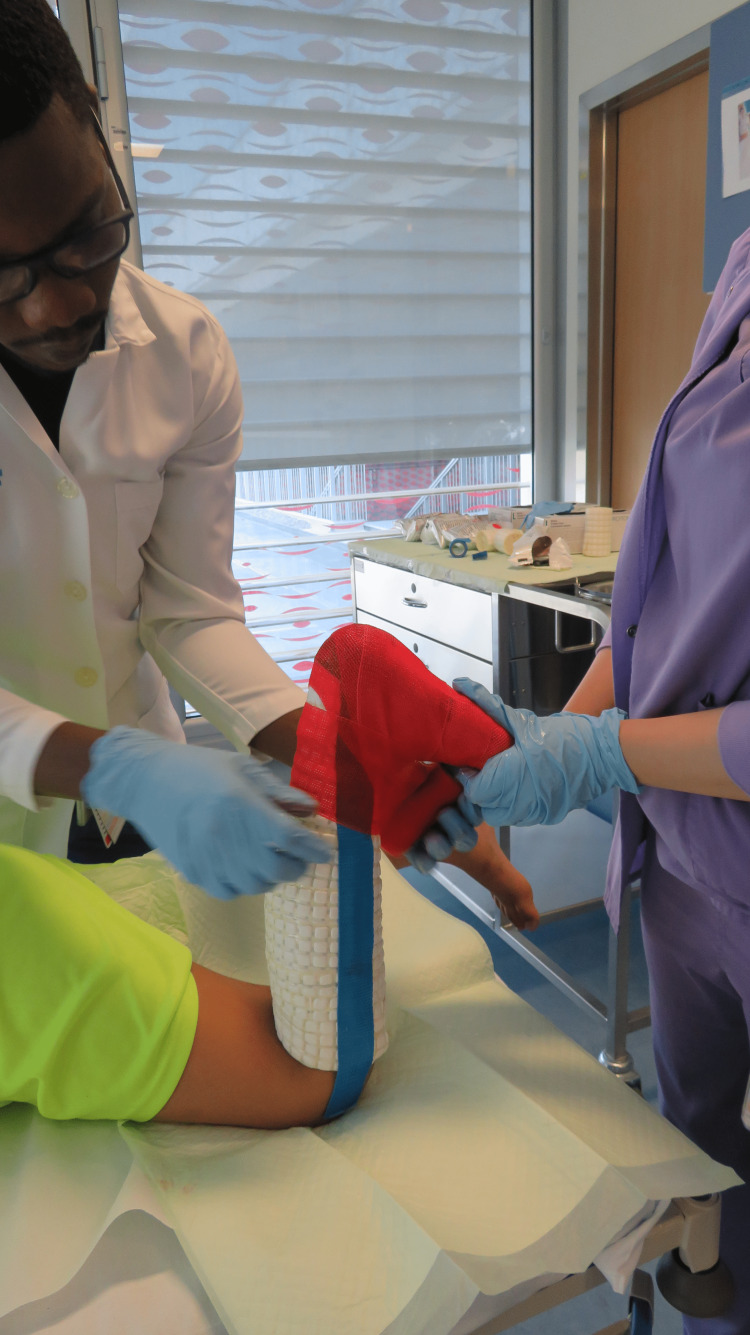
Clinical photograph showing an effective way of performing the serial casting Serial casting involves bending the knee joint to 90° to relax the gastrocnemius muscles; then casting the ankle in maximum dorsiflexion. The gastrocnemius muscles get stretched when the knee joint becomes straight during walking. The image is provided by the authors.

The second line of treatment is serial casting, when gastrocnemius muscles are stretched on a weekly basis by casting the ankle in maximum dorsiflexion (Figure 10). Serial casting involves bending the knee joint to 90° to relax the gastrocnemius muscles; then casting the ankle in maximum dorsiflexion. The gastrocnemius muscles get stretched when the knee joint becomes straight during walking. It is less effective in treating soleus muscle tightness or tendoachillis tightness. It also does not work if the treated child does not walk.

Both non-operative treatments (physiotherapy and serial casting) require a rigid foot to be used as a lever to stretch the tight gastrocnemius muscles. Unfortunately, the foot rigidity declines when the talonavicular joint starts subluxing, which makes muscles stretching difficult, if not impossible. Moreover, using excessive force to stretch gastrocnemius muscles during physiotherapy or serial casting could accelerate the talonavicular subluxation, and careful considerations in borderline cases are important.

**Figure 11:**
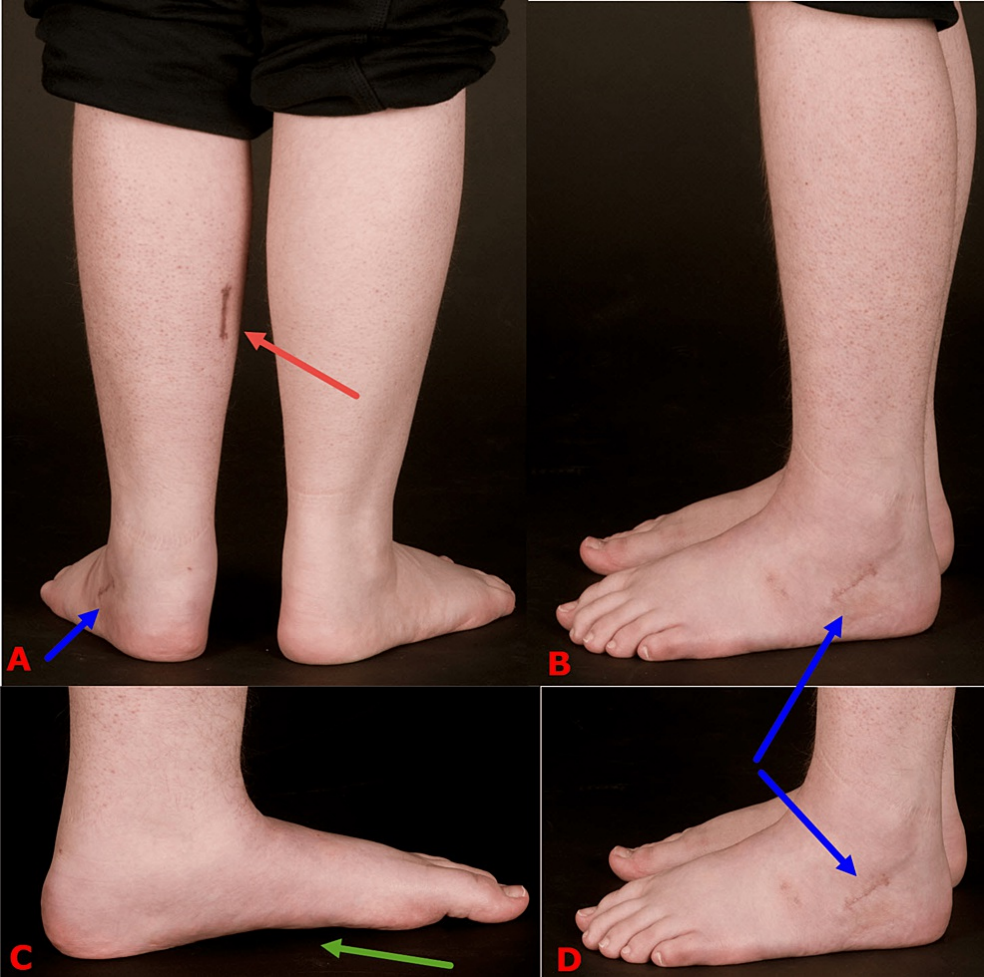
Clinical photographs showing the postoperative correction Clinical photographs following the surgical correction of the same child that is shown in Figure 3. The tight gastrocnemius muscle is lengthened through a small incision on the back of the leg (solid red arrow on image A). The calcaneus bone is lengthened using a small incision on the outside of the foot (solid blue arrows on images B and D). The medial longitudinal arch is restored (solid green arrow on image C). Images are provided by the authors.

**Figure 12:**
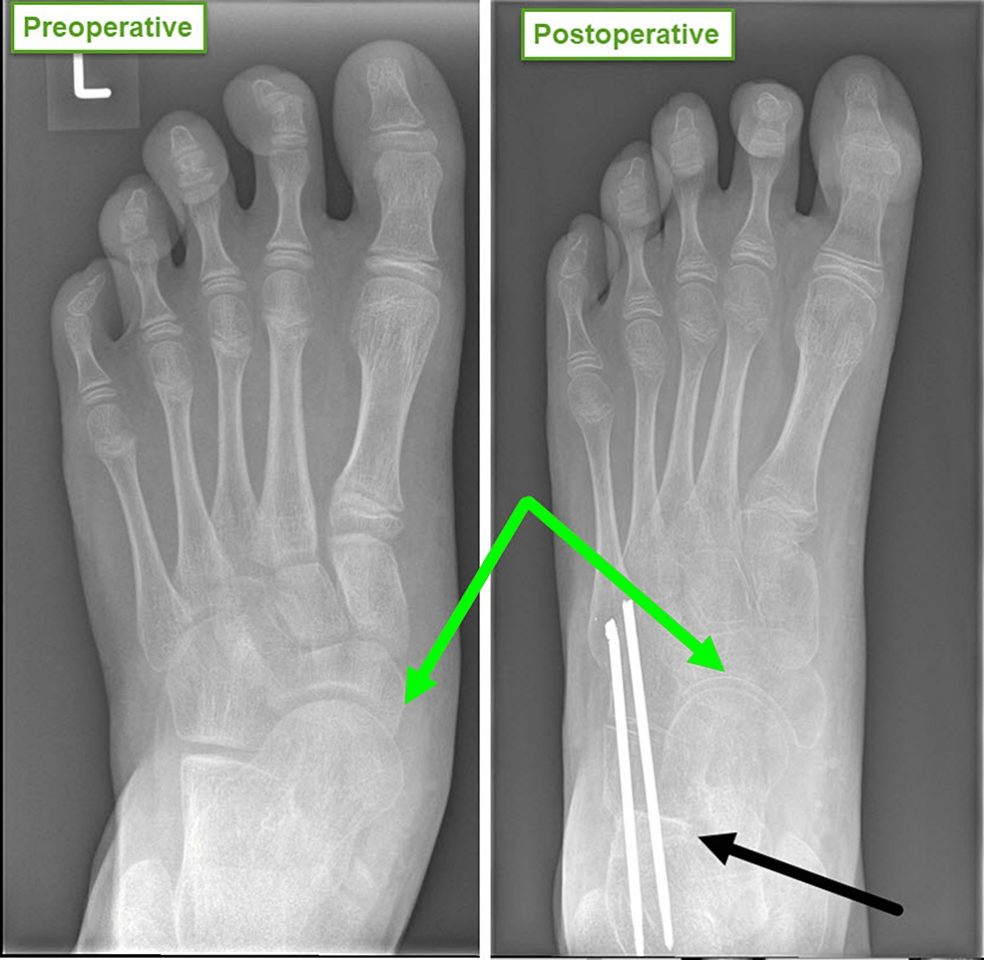
Postoperative plain radiograph Plain foot radiographs before and after surgery show the relocation of the talonavicular joint by lengthening the calcaneus bone using a wedge bone graft (black arrow). Images are provided by the authors.

The third line of treatment is surgical correction. This typically involves three procedures (Figure 11). First, a fractional lengthening of the gastrocnemius muscles through a small incision on the back of the leg. Second, acute calcaneal lengthening using osteotomy and wedge bone graft to reduce the talonavicular joint. The stretched capsule of the joint often shrinks with time when the joint is reduced. In severe cases, we surgically shrink and strengthen the capsule using the double-breasting technique. The bone graft can be artificial, allograft or autograft. Historically, we preferred autograft in children who are over eight years old, fearing calcaneal osteotomy nonunion. However, the quality of artificial graft has improved significantly and has become our first choice for all patients. Third, the foot is protected in a short cast for a period of six weeks, when a plain radiograph of the foot is obtained to confirm bony cuts healing and the accuracy of correction (Figure 12). This is even more important when an artificial bone graft is used. Patients can start weight bearing fully with the aid of physiotherapists after six weeks from the date of surgery.

## Conclusions

Lifestyle is changing at a very fast pace, often in the right direction but not always. We need to remain watchful for newly emerging health disorders and raise awareness. The number of children with muscles tightness and adolescent-acquired flatfeet has risen astronomically over the last decade. This has even become worse with the COVID-19 pandemic and subsequent lockdown. This condition develops and worsens silently. Initially, there are no symptoms. Signs are inconspicuous, even for the most vigilant parents. Therefore, most affected children sadly present late during the course of the condition when simple non-operative treatments, such as physiotherapy and casting, become ineffective. Although surgical intervention is very successful in correcting the condition, it is completely avoidable. Prevention is better than cure, and adolescent-acquired flatfeet is a fully preventable condition using simple measures. However, public-wide awareness is paramount.
